# Supplementary material for: Initiating a watch list for Ebola virus antibody escape mutations
Source: PeerJ. 2016 Feb 16;4:e1674. doi: 10.7717/peerj.1674 (PMC4768679; doi:10.7717/peerj.1674)
Supplement: Supplemental Information 1 [file peerj-04-1674-s001.pdf]

Supplemental Materials: Initiating a watch list for Ebola Virus Antibody Escape Mutations

CONTENTS

1	Overview	1
2	Workflow	1
3	Selection of Test Systems	1
4	Preparation of Structures	2
5	Molecular Dynamics Simulations	2
6	FoldX Analysis	2
7	Statistical Analysis	2
8	Results	2
References		7

1 OVERVIEW

We hypothesized that because protein structures are not static, improvements in $\Delta\Delta G$ estimation might be achieved by using molecular dynamics (MD) simulation to sample the configurational space for the proteins and then analyze snapshots from these simulations in FoldX. To test this strategy, we selected 20 test systems (10 folding and 10 binding) for which experimental structures and sufficient experimental data were available. For each system we estimated $\Delta\Delta G$ using FoldX software either applied to the experimental structure directly (the conventional approach) or by first using MD simulation to sample 100 snapshots, processing each with FoldX, and then averaging (the proposed strategy). We then compared the ability of the two approach to predict experimental data and found averaging over MD snapshots improves estimation. Below we provide details of our methods and results.

2 WORKFLOW

The code, scripts, and input files that execute all the work described here and in the main manuscript are contained in a folder that accompanies this document. In this folder, the organizing ”read me” file is called called ”Workflow Notebook.html”. This was created using as an R Jupyter Notebook. The notebook file is also provided (Workflow Notebook.ipynb). This notebook contains all the details including descriptions of every file and the R code used in the analyses.

3 SELECTION OF TEST SYSTEMS

To provide a test of our ability to predict folding and binding stabilities we selected 20 model systems. For folding we selected ten systems from the ProTherm database (Kumar et al., 2006) and for binding we selected ten systems from the Skempi database (Moal and Fernández-Recio, 2012). The criteria for selecting the systems were: (i) the values for $\Delta\Delta G$ were varied in sign since negative, stabilizing values are typically harder to predict than positive, destabilizing values, (ii) there were more than 20 experimental point mutations, and not all were mutations to alanine, (iii) the structures in the PDB were not missing a large number of residues and (iv) only one system was chosen in cases where the proteins were the same but had different protein databank (PDB) structures. The PDB identifiers selected for our test systems can be seen in Supplemental Figs 1 and 2. Two of the ten binding test systems (2jel, 3hfm) contain antibody-epitope interactions and thus are especially relevant to the current study.

4 PREPARATION OF STRUCTURES

Each test system was prepared in an identical manner using the following steps.

1. Experimental structures were downloaded from the PDB website.
2. The structure files were edited to remove all but the necessary chain(s).
3. Any residues that were missing in the PDB files were fixed using MODELLER (Sali and Blundell, 1993).
4. Each structure was processed using pdb2pqr to ensure that the nomenclature was standardized (Dolinsky et al., 2004).

5 MOLECULAR DYNAMICS SIMULATIONS

The *Molecular Dynamics Simulations* subsection within *Methods* in the main manuscript describes how MD simulations were used to obtain 100 snapshots.

6 FOLDX ANALYSIS

FoldX was used to estimate $\Delta\Delta G$ values using two approaches.

1. **Experimental Structure (Ex):** FoldX was used to analyze a single experimental PDB structure. A single set of $\Delta\Delta G$ values was generated (one value for each mutation).
2. **MD Simulations (MD):** MD simulations were used to generate 100 snapshots (PDB structures). Each of these structures was analyzed using FoldX, generating 100 sets of $\Delta\Delta G$ values. For each mutation, the 100 $\Delta\Delta G$ values were averaged to obtain the final estimate for $\Delta\Delta G$.

The way that FoldX was used on the experimental structure or on the MD generated structures is described in the main paper in the *FoldX* subsection within Methods.

7 STATISTICAL ANALYSIS

For each of the 20 systems, we created a scatterplot of empirically estimated $\Delta\Delta G$ values vs (i) predicted $\Delta\Delta G$ based on experimental structure (Ex) method and (ii) predicted $\Delta\Delta G$ based on the MD simulation method (MD). Using the R statistical language (R Core Team, 2015), we calculated bias and root mean squared error for each method of prediction in each system. For a given test system, bias and root mean squared error (rMSE) are the average distance and average absolute distance, respectively, between the predicted and observed values across all mutations. For each system, we also performed a simple linear regression between predicted and observed $\Delta\Delta G$ values and summarized the fit using R^2.

8 RESULTS

The test system results indicate that the MD simulation method improves the ability to predict $\Delta\Delta G$ for both folding and binding over the Ex method. The scatterplots, regression lines and R^2 values for the 10 folding and 10 binding systems are presented in Supplemental Figs 1 and 2, respectively. Supplemental Tables 1 and 2 provide, for folding and binding, respectively, the bias, rMSE, and summary of regression results (intercept, slope, R^2) for each system along with averages across all 10 systems.

For folding, the MD method is, on average, less biased than the Ex method (0.08 vs 0.40, respectively), has lower rMSE (1.13 vs 1.37) and has higher R^2 values (0.34 vs 0.22) (Supplemental Table 1). We can also examine performance on individual test systems by the same criteria. First removing instances where the two methods are essentially equally good (within 0.03 of each other), we find that MD has lower bias than Ex in 5 of 9 systems, lower rMSE in 9 of 10 systems, and higher R^2 in 7 of 8 systems. The results for binding are very similar. The MD strategy again has lower average bias than Ex (0.38 vs 0.40), lower average rMSE (1.17 vs 1.36) and higher R^2 (0.35 vs 0.27) (Supplemental Table 2). At the level of individual test systems for binding, MD has lower bias than Ex in 8 or 9 systems, lower rMSE in 9 of 9 systems, and higher R^2 in 6 of 8 systems.

Based on these results we conclude that the MD strategy introduced here marks a substantial improvement over Ex in estimating $\Delta\Delta G$ for both folding and binding. We therefore employ this strategy on the Ebola system.

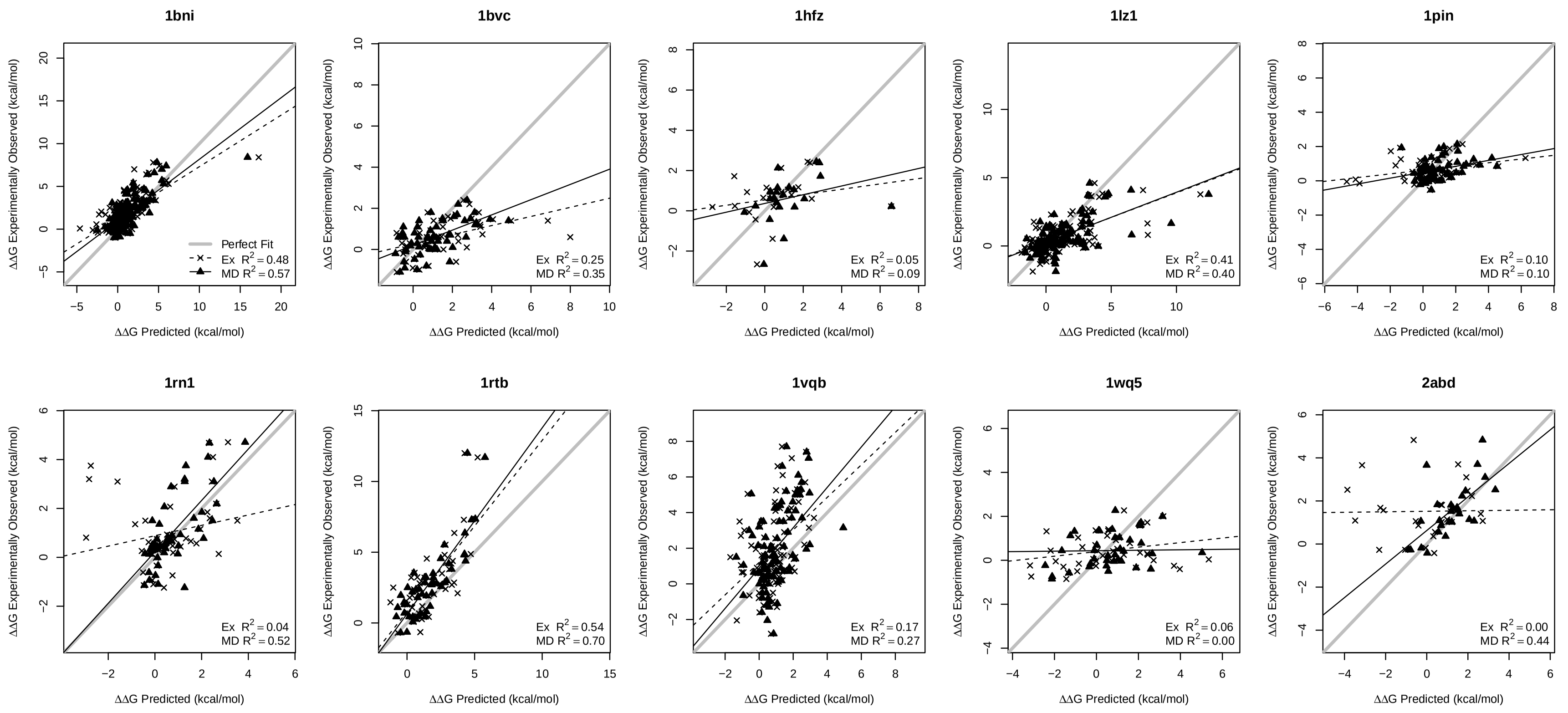

Figure 1. **Predicted folding $\Delta\Delta G$ vs experimentally observed values in 10 protein systems.** System names (PDB IDs) are given above each panel. The two methods of prediction are (1) using FoldX on the experimental structure (Ex) and (2) using FoldX on each of 100 samples taken from a MD simulation and averaging (MD). See methods for details. Perfect fit data would fall along the gray diagonal line. The solid and dashed black lines show the linear relationship between prediction and observation with method of prediction (Ex or MD) and corresponding R^2 values given in the inset legends.

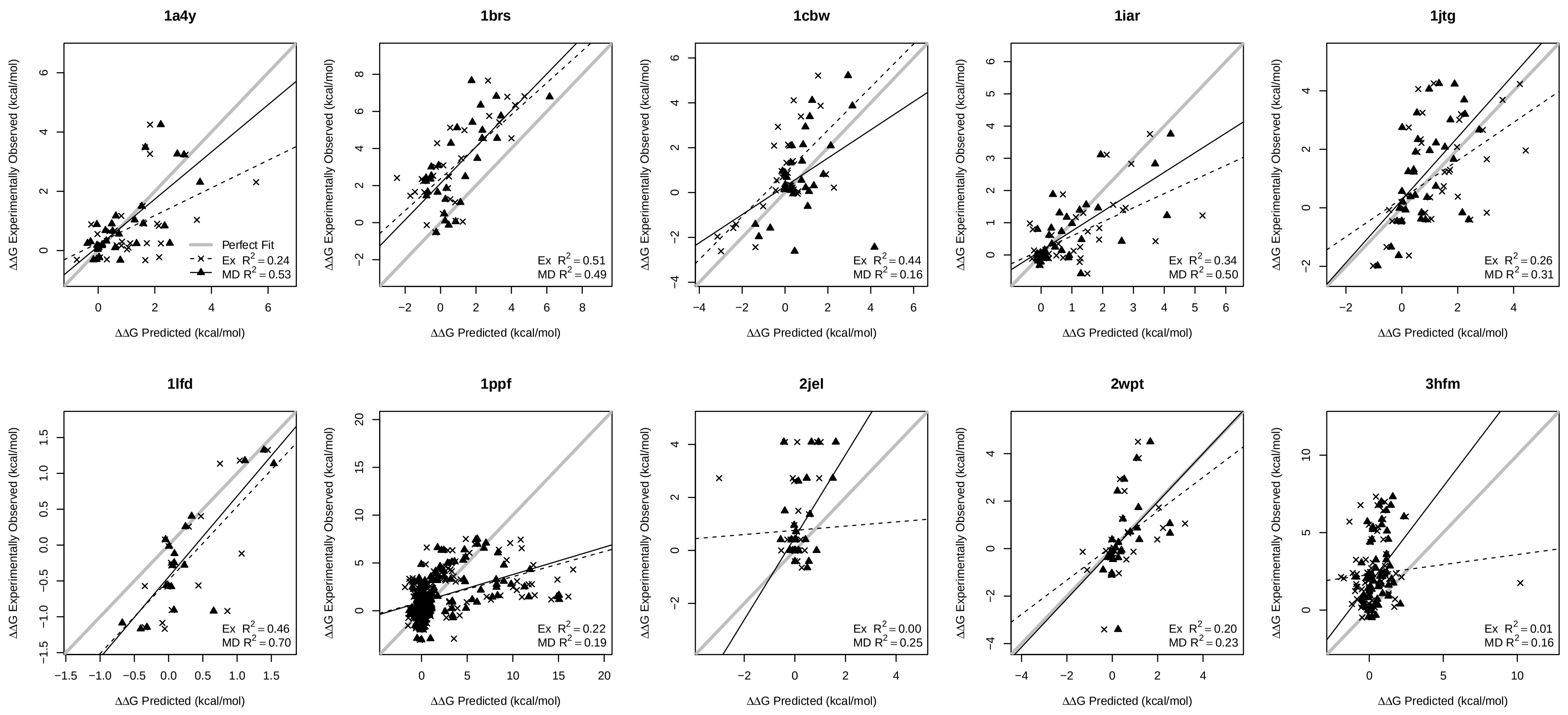

Figure 2. Predicted binding $\Delta\Delta G$ vs experimentally observed values in 10 protein systems. System names (PDB IDs) are given above each panel. The two methods of prediction are (1) using FoldX on the experimental structure (Ex) and (2) using FoldX on each of 100 samples taken from a MD simulation and averaging (MD). See methods for details. Perfect fit data would fall along the gray diagonal line. The solid and dashed black lines show the linear relationship between prediction and observation with method of prediction (Ex or MD) and corresponding R^2 values given in the inset legends.

Protein	# Residues	# Mutations	$Bias_{Ex}$	$rMSE_{Ex}$	Int_{Ex}	$Slope_{Ex}$	R^2_{Ex}	$Bias_{MD}$	$rMSE_{MD}$	Int_{MD}	$Slope_{MD}$	R^2_{MD}
1bni	67	163	0.95	1.31	1.29	0.60	0.48	0.66	1.02	0.98	0.72	0.57
1bvc	37	56	-0.85	1.20	0.27	0.22	0.25	-0.47	0.88	0.20	0.37	0.35
1hfz	12	23	0.04	1.26	0.54	0.13	0.05	-0.54	1.04	0.37	0.22	0.09
1lz1	53	116	-0.43	1.12	0.29	0.36	0.41	-0.48	1.05	0.26	0.37	0.40
1pin	32	56	0.23	1.18	0.62	0.11	0.10	-0.30	0.80	0.50	0.17	0.10
1rn1	24	49	0.47	1.18	0.88	0.21	0.04	0.26	0.77	0.22	1.05	0.52
1rtb	22	50	1.15	1.50	0.79	1.21	0.54	1.17	1.34	0.64	1.31	0.70
1vqb	34	92	1.13	1.79	1.20	0.91	0.17	1.01	1.69	0.88	1.13	0.27
1wq5	13	43	-0.13	1.35	0.39	0.10	0.06	-1.13	1.99	0.44	0.01	0.00
2abd	20	27	1.48	1.78	1.52	0.01	0.00	0.37	0.70	0.63	0.78	0.44
Means	31	66	0.40	1.37	0.78	0.40	0.22	0.08	1.13	0.52	0.64	0.34

Table 1. Summary of fit between two methods of prediction (Ex and MD) and empirically observed values of folding stability in 10 protein systems. # Residues is less than # Mutations because experiments involved multiple amino acid changes at the same residue. Other column headers: ‘Bias’ and ‘rMSE are, respectively, the the average distance and the average absolute distance between predicted and observed $\Delta\Delta G$ values in kcal/mol; ‘Int’ and ‘Slope’ are the intercept and slope of the best fit linear regression line.

Protein	# Residues	# Mutations	$Bias_{Ex}$	$rMSE_{Ex}$	Int_{Ex}	$Slope_{Ex}$	R^2_{Ex}	$Bias_{MD}$	$rMSE_{MD}$	Int_{MD}	$Slope_{MD}$	R^2_{MD}
1a4y	29	32	-0.41	0.97	0.25	0.47	0.24	-0.03	0.58	0.14	0.80	0.53
1brs	17	30	2.26	2.40	2.37	0.86	0.51	2.11	2.23	2.12	0.98	0.49
1cbw	15	31	0.86	1.24	0.86	0.96	0.44	-0.00	1.16	0.28	0.63	0.16
1iar	20	36	-0.50	0.82	0.15	0.44	0.34	-0.21	0.55	0.13	0.61	0.50
1jtg	35	37	-0.12	1.16	0.31	0.65	0.26	0.32	1.12	0.27	1.07	0.31
1lfd	17	19	-0.48	0.55	-0.49	1.03	0.46	-0.42	0.44	-0.44	1.13	0.70
1ppf	10	190	-0.36	2.09	0.94	0.26	0.22	-0.27	1.89	0.92	0.29	0.19
2jel	32	43	0.75	0.87	0.76	0.08	0.00	0.59	0.76	0.50	1.56	0.25
2wpt	22	26	-0.02	1.05	0.12	0.72	0.20	-0.06	0.98	-0.07	1.02	0.23
3hfm	21	71	2.07	2.45	2.29	0.13	0.01	1.81	1.95	1.69	1.26	0.16
Means	22	52	0.40	1.36	0.76	0.56	0.27	0.38	1.17	0.55	0.94	0.35

Table 2. Summary of fit between two methods of prediction (Ex and MD) and empirically observed values of binding stability in 10 protein systems. # Residues is less than # Mutations because experiments involved multiple amino acid changes at the same residue. Other column headers: 'Bias' and 'rMSE are, respectively, the the average distance and the average absolute distance between predicted and observed $\Delta\Delta G$ values in kcal/mol; 'Int' and 'Slope' are the intercept and slope of the best fit linear regression line; R^2 is the proportion of variation explained by the linear regression.

REFERENCES

Dolinsky, T. J., Nielsen, J. E., McCammon, J. A., and Baker, N. A. (2004). PDB2PQR: an automated pipeline for the setup of poisson-boltzmann electrostatics calculations. *Nucleic Acids Research*, 32(Web Server):W665–W667.

Kumar, M. S., Bava, K. A., Gromiha, M. M., Prabakaran, P., Kitajima, K., Uedaira, H., and Sarai, A. (2006). Protherm and pronit: thermodynamic databases for proteins and protein–nucleic acid interactions. *Nucleic Acids Research*, 34(suppl 1):D204–D206.

Moal, I. H. and Fernández-Recio, J. (2012). Skempi: a structural kinetic and energetic database of mutant protein interactions and its use in empirical models. *Bioinformatics*, 28(20):2600–2607.

R Core Team (2015). *R: A Language and Environment for Statistical Computing*. R Foundation for Statistical Computing, Vienna, Austria.

Sali, A. and Blundell, T. L. (1993). Comparative protein modelling by satisfaction of spatial restraints. *Journal of Molecular Biology*, 234(3):779–815.
