# Supplementary material for: Initiating a watch list for Ebola virus antibody escape mutations
Source: PeerJ. 2016 Feb 16;4:e1674. doi: 10.7717/peerj.1674 (PMC4768679; doi:10.7717/peerj.1674)
Supplement: Supplemental Information 2 [file peerj-04-1674-s002.html]

Workflow Notebook


# Notebook of Workflow for "Initiating a watch list for Ebola virus antibody escape mutations"¶

The notebook provides details of the analysis including scripts, code, and file descriptions.

## Table of Conents¶

- Molecular Dynamics Simulations
- FoldX
- GROMACS
- R Analysis of Test Systems:
  - Folding
  - Binding
- R Anlaysis of Ebola System

## Molecular Dynamics Simulations¶

#### Structure files used in MD simulations.¶

- ebov\_gp1.pdb -- GP1 monomer structure used as input for molecular dynamics (MD)
- ebov\_gp2.pdb -- GP2 monomer structure used as input for MD
- ebov\_gp1-gp2.pdb -- trimer of GP1-GP2 dimers used as input for MD
- ebov\_gp1-gp2-ab.pdb -- trimer of GP1-GP2-KZ52

#### MD parameter files¶

- em.mdp -- Gromacs input file for energy minimization
- pr.mdp -- Gromacs input file for position restrained equilibration
- md.mdp -- Gromacs input file for unrestrained equilibration
- prod.mdp -- Gromacs input file for production simulations used to generate frames for FoldX

## FoldX¶

#### Scripts¶

- foldx\_scan.py -- Python script used to run FoldX on a computer cluster  
  Folding stability command example: 'foldx\_scan.py -i wt.pdb -n 10 -t 300 -p'  
  Binding stability command example: 'foldx\_scan.py -i wt.pdb -n 20 -g ABC -m ABC -t 300 -p'
- pdb2pqr.sh -- Bash script used to process files using pdb2pqr  
  Command example: 'pdb2pqr.sh -i in -s'

## GROMACS Commands¶

The following list of commands uses structure 'in.pdb'. The same list was used for all molecular dynamics simulations.

gmx-5.0 pdb2gmx -vsite hydrogens -ff charmm22star -water tip3p -ignh -f in.pdb -o out.gro -p out.top -i out.posre  
gmx-5.0 editconf -f out.gro -o out.gro -bt dodecahedron -d 1.0  
gmx-5.0 solvate -cs -cp out.gro -o out.gro -p out.top  
gmx-5.0 grompp -f em -po em.mdout -c out.gro -p out.top -o em  
gmx-5.0 genion -s em -o out.gro -p out.top -conc 0.15 -neutral -pname NA -nname CL  
gmx-5.0 grompp -f em -po em.mdout -c out.gro -p out.top -o em  
mdrun-5.0\_gpu -ntomp 0 -pin on -v -cpo pr -s pr -o pr -x pr -c pr.last -e pr -g pr  
gmx-5.0 grompp -f pr -po pr.mdout -c em.last -p out.top -o pr  
mdrun-5.0\_gpu -ntomp 0 -pin on -v -cpo pr -s pr -o pr -x pr -c pr.last -e pr -g pr  
gmx-5.0 grompp -f md -po md.mdout -c pr.last -p out.top -o md  
mdrun-5.0\_gpu -ntomp 0 -pin on -v -cpo md -s md -o md -x md -c md.last -e md -g md  
gmx-5.0 grompp -f prod -po prod.mdout -c out -p out -o prod  
mdrun-5.0\_gpu -ntomp 0 -pin on -cpo prod -s prod -o prod -x prod -c prod.last -e prod -g prod

## R Analysis of Test Systems¶

The folder 'test\_systems' holds the experiemtnal and predicted $\Delta \Delta G$ values for each of the 10 folding and the 10 binding systems. These 20 files all have same format, each wiht the following four columns: (1) mutation (`mut#`); (2) experimentally determined ddG value (`exp`); (3) predcited ddG values using FoldX applied only to the experimental structure without MD simulations (`foldx_exp`); (4) predicted $\Delta \Delta G$ values based on our method of anlayzing each of 100 MD snapshots with FoldX and then averaging. The following R code was used to read in and compare the abiltiy of two methods of predicting stability effects.

In [142]:

```
# Locate and input test system results

setwd("/Users/Craig/Dropbox/Craig_Work/Project--Ebola/Jupytr/Workflow_Files/test_systems")
files <- list.files()
exp.colnames <- c("mutation", "exp.ddG", "foldX.ddG", "MD.fX.ddG")

file.names.list <- sapply(files, function(x) strsplit(x, split="_"))
fold.files <- which(unlist(lapply(file.names.list, function(x) x[2] == "fold")))
bind.files <- which(unlist(lapply(file.names.list, function(x) x[2] == "bind")))
    
fold.systems1 <- sapply(names(fold.files), function(x) unlist(strsplit(x, split="_"))[3])
fold.systems2 <- sapply(fold.systems1, function(x) unlist(strsplit(x, split="\\."))[1])
bind.systems1 <- sapply(names(bind.files), function(x) unlist(strsplit(x, split="_"))[3])
bind.systems2 <- sapply(bind.systems1, function(x) unlist(strsplit(x, split="\\."))[1])

systems2 <- fold.systems2
    
system.names <- lapply(file.names.list, function(x) unlist(strsplit(x[3], split="\\."))[1])
n.systems <- length(fold.files)
```

###### Analysis of Foldling Systems¶

First cell below defines whether to output pdf and table reslts.
Second cell below does analysis and creates outputs as specified.

In [143]:

```
plot.pdf <- TRUE
pdf.file <- "Folding ddG experimental systems (v2).pdf"
print.table <- TRUE
table.file <- "Folding Summary Table v2.txt"
```

In [144]:

```
files.to.use <- fold.files

# Set to TRUE if you want to produce the pdf
plot.pdf <- TRUE

if (plot.pdf == TRUE){
    pdf(file="Folding ddG experimental systems (v2).pdf", width=15, height=7)
    layout(mat=matrix(nrow=2, ncol=5, data=seq(1,10), byrow=TRUE), heights=rep(3,2), widths=rep(3,5)) -> l
    #layout.show(l)
    #dev.off()
}
    
# Analysis 
lm.smry <- as.data.frame(matrix(nrow=length(systems2)+1, ncol=13))
colnames(lm.smry) <- c("Protein", "n.sites", "n.mutations", "FX.bias", "FX.rMSE", "FX.Intercept", "FX.Slope", "FX.R2", "MDS.bias", "MDS.rMSE", "MDS.Intercept", "MDS.Slope", "MDS.R2")
lm.smry$Protein <- c(systems2, "Means")
round.vals <- c(NA, NA, 0,0, 2,2,2,2,2,2,2,2,2,2)
    
    for (file.i in 1:length(files.to.use)){
        data <- read.table(file=files[files.to.use[file.i]])
        colnames(data) <- exp.colnames
        max.E <- max(data$exp.ddG)
        max.F <- max(data$foldX.ddG)
        max.M <- max(data$MD.fX.ddG)
        min.E <- min(data$exp.ddG)
        min.F <- min(data$foldX.ddG)
        min.b <- min(min.E, min.F)
        max.b <- max(max.E, max.F)
        
        lm <- lm(data$exp.ddG ~ data$foldX.ddG)
        lm2 <- lm(data$exp.ddG ~ data$MD.fX.ddG )

        mean.error.FX <- mean(abs(data$exp.ddG - data$foldX.ddG))
        mean.error.MDS <- mean(abs(data$exp.ddG - data$MD.fX.ddG))
        bias.FX <- mean(data$exp.ddG - data$foldX.ddG)
        bias.MDS <- mean(data$exp.ddG - data$MD.fX.ddG)
        
        muts <- as.character(data$mutation)
        sites <- sapply(muts, function(x) substring(x, 3, nchar(x)-1))
        
        lm.smry$n.sites[file.i] <- length(unique(sites))
        lm.smry$n.mutations[file.i]<- length(muts)
        lm.smry$FX.Intercept[file.i] <- round(coefficients(lm)[1],2)
        lm.smry$FX.Slope[file.i] <- round(coefficients(lm)[2],2)
        lm.smry$FX.R2[file.i] <- round(summary(lm)$r.squared,2)
        lm.smry$FX.rMSE[file.i] <- round(mean.error.FX,2)
        lm.smry$MDS.Intercept[file.i] <- round(coefficients(lm2)[1],2)
        lm.smry$MDS.Slope[file.i] <- round(coefficients(lm2)[2],2)
        lm.smry$MDS.R2[file.i] <- round(summary(lm2)$r.squared,2)
        lm.smry$MDS.rMSE[file.i] <- round(mean.error.MDS,2)
        lm.smry$FX.bias[file.i] <- round(bias.FX,2)
        lm.smry$MDS.bias[file.i] <- round(bias.MDS,2)
        
        mean.exp <- mean(data$exp.ddG)
        TSS <- sum((data$exp.ddG - mean.exp)^2)
        
        fX.SS <- sum((data$exp.ddG - data$foldX.ddG)^2)
        fX.Resids <- (data$exp.ddG - data$foldX.ddG)
        MS.SS <- sum((data$exp.ddG - data$MD.fX.ddG)^2)
        MS.resids <- (data$exp.ddG - data$MD.fX.ddG)
        
        R2.fX <- (TSS - fX.SS)/TSS
        R2.MS <- (TSS - MS.SS)/TSS
            
        if (plot.pdf == TRUE){
            par(mar=c(5,5,4,1))
            grey.col <- "grey75"
            pt.cex <- 0.8
            leg.loc <- "bottomright"
            scale <- 1.2
            plot(data$MD.fX.ddG, data$exp.ddG, pch=24, bg="black", cex=pt.cex, xlim=c(min.b*scale, max.b*scale), ylim=c(min.b*scale, max.b*scale), xlab=expression(paste(Delta, Delta, G, " Predicted", " (kcal/mol)")), ylab=expression(paste(Delta, Delta, G, " Experimentally Observed", " (kcal/mol)")), main=systems2[file.i])
            abline(0,1, lty="solid", col=grey.col, lwd=3)
            points(data$foldX.ddG, data$exp.ddG, pch=4, bg="white", cex=pt.cex)
            abline(coefficients(lm), lty="dashed")
            abline(coefficients(lm2))
            points(data$foldX.ddG, data$exp.ddG, pch=4, bg="black")
            points(data$MD.fX.ddG, data$exp.ddG, pch=24, bg="black")
            if (file.i == 1){
                legend(leg.loc, legend=c("Perfect Fit", as.expression(bquote(paste("Ex  ", R^2==.(format(round(summary(lm)$r.squared,2), nsmall=2))))), as.expression(bquote(paste("MD ", R^2==.(format(round(summary(lm2)$r.squared,2), nsmall=2)))))), bty="n", lty=c("solid", "dashed", "solid"), col=c(grey.col,"black", "black"), pch=c(NA, 4, 24), pt.bg=c(NA, NA, "black"), lwd=c(3,1,1))
           } else{
                legend(leg.loc, legend=c(as.expression(bquote(paste("Ex  ", R^2==.(format(round(summary(lm)$r.squared,2), nsmall=2))))), as.expression(bquote(paste("MD ", R^2==.(format(round(summary(lm2)$r.squared,2),nsmall=2)))))), bty="n")
           }
        }
    }
                        
    for (col.i in 2:length(lm.smry[1,])){
        colmean <- round(mean(lm.smry[,col.i], na.rm=TRUE), round.vals[col.i])
        lm.smry[n.systems+1,col.i] <- colmean
    }
    
    lm.smry
                        
    if (plot.pdf == TRUE){
        dev.off()
    }
                        
    if (print.table == TRUE){
        write.table(x=lm.smry, file=table.file, col.names=TRUE, row.names=FALSE, sep="\t")
    }
    
    # Unpound these lines to output the results as LaTex Table
        #library(xtable)
        #x <- xtable(lm.smry, digits=c(1,round.vals), floating=FALSE, tabular.environment="tabular", hline.after=NULL, include.rownames=FALSE, include.colnames=TRUE, caption="Folding Stability")
        #print(x, include.rownames=FALSE)
```

Out[144]:

|  | Protein | n.sites | n.mutations | FX.bias | FX.rMSE | FX.Intercept | FX.Slope | FX.R2 | MDS.bias | MDS.rMSE | MDS.Intercept | MDS.Slope | MDS.R2 |
| --- | --- | --- | --- | --- | --- | --- | --- | --- | --- | --- | --- | --- | --- |
| 1 | 1bni | 67 | 163 | 0.95 | 1.31 | 1.29 | 0.6 | 0.48 | 0.66 | 1.02 | 0.98 | 0.72 | 0.57 |
| 2 | 1bvc | 37 | 56 | -0.85 | 1.2 | 0.27 | 0.22 | 0.25 | -0.47 | 0.88 | 0.2 | 0.37 | 0.35 |
| 3 | 1hfz | 12 | 23 | 0.04 | 1.26 | 0.54 | 0.13 | 0.05 | -0.54 | 1.04 | 0.37 | 0.22 | 0.09 |
| 4 | 1lz1 | 53 | 116 | -0.43 | 1.12 | 0.29 | 0.36 | 0.41 | -0.48 | 1.05 | 0.26 | 0.37 | 0.4 |
| 5 | 1pin | 32 | 56 | 0.23 | 1.18 | 0.62 | 0.11 | 0.1 | -0.3 | 0.8 | 0.5 | 0.17 | 0.1 |
| 6 | 1rn1 | 24 | 49 | 0.47 | 1.18 | 0.88 | 0.21 | 0.04 | 0.26 | 0.77 | 0.22 | 1.05 | 0.52 |
| 7 | 1rtb | 22 | 50 | 1.15 | 1.5 | 0.79 | 1.21 | 0.54 | 1.17 | 1.34 | 0.64 | 1.31 | 0.7 |
| 8 | 1vqb | 34 | 92 | 1.13 | 1.79 | 1.2 | 0.91 | 0.17 | 1.01 | 1.69 | 0.88 | 1.13 | 0.27 |
| 9 | 1wq5 | 13 | 43 | -0.13 | 1.35 | 0.39 | 0.1 | 0.06 | -1.13 | 1.99 | 0.44 | 0.01 | 0 |
| 10 | 2abd | 20 | 27 | 1.48 | 1.78 | 1.52 | 0.01 | 0 | 0.37 | 0.7 | 0.63 | 0.78 | 0.44 |
| 11 | Means | NA | 68 | 0 | 1.37 | 0.78 | 0.39 | 0.21 | 0.06 | 1.13 | 0.51 | 0.61 | 0.34 |

Out[144]:

**pdf:** 2

###### Analysis of Binding Systems¶

First cell below defines whether to output pdf and table reslts.
Second cell below does analysis and creates outputs as specified.

In [145]:

```
plot.pdf <- TRUE
pdf.file <- "Binding ddG experimental systems (v2).pdf"
print.table <- TRUE
table.file <- "Binding Summary Table v2.txt"
```

In [146]:

```
# Note that this code is the duplicated from the folding code above.

files.to.use <- bind.files

if (plot.pdf == TRUE){
    pdf(file=pdf.file, width=15, height=7)
    layout(mat=matrix(nrow=2, ncol=5, data=seq(1,10), byrow=TRUE), heights=rep(3,2), widths=rep(3,5)) -> l
    #layout.show(l)
    #dev.off()
}
    
# Analysis 
lm.smry <- as.data.frame(matrix(nrow=length(systems2)+1, ncol=13))
colnames(lm.smry) <- c("Protein", "n.sites", "n.mutations", "FX.bias", "FX.rMSE", "FX.Intercept", "FX.Slope", "FX.R2", "MDS.bias", "MDS.rMSE", "MDS.Intercept", "MDS.Slope", "MDS.R2")
lm.smry$Protein <- c(systems2, "Means")
round.vals <- c(NA, NA, 0,0, 2,2,2,2,2,2,2,2,2,2)
    
    for (file.i in 1:length(files.to.use)){
        data <- read.table(file=files[files.to.use[file.i]])
        colnames(data) <- exp.colnames
        max.E <- max(data$exp.ddG)
        max.F <- max(data$foldX.ddG)
        max.M <- max(data$MD.fX.ddG)
        min.E <- min(data$exp.ddG)
        min.F <- min(data$foldX.ddG)
        min.b <- min(min.E, min.F)
        max.b <- max(max.E, max.F)
        
        lm <- lm(data$exp.ddG ~ data$foldX.ddG)
        lm2 <- lm(data$exp.ddG ~ data$MD.fX.ddG )

        mean.error.FX <- mean(abs(data$exp.ddG - data$foldX.ddG))
        mean.error.MDS <- mean(abs(data$exp.ddG - data$MD.fX.ddG))
        bias.FX <- mean(data$exp.ddG - data$foldX.ddG)
        bias.MDS <- mean(data$exp.ddG - data$MD.fX.ddG)
        
        muts <- as.character(data$mutation)
        sites <- sapply(muts, function(x) substring(x, 3, nchar(x)-1))
        
        lm.smry$n.sites[file.i] <- length(unique(sites))
        lm.smry$n.mutations[file.i]<- length(muts)
        lm.smry$FX.Intercept[file.i] <- round(coefficients(lm)[1],2)
        lm.smry$FX.Slope[file.i] <- round(coefficients(lm)[2],2)
        lm.smry$FX.R2[file.i] <- round(summary(lm)$r.squared,2)
        lm.smry$FX.rMSE[file.i] <- round(mean.error.FX,2)
        lm.smry$MDS.Intercept[file.i] <- round(coefficients(lm2)[1],2)
        lm.smry$MDS.Slope[file.i] <- round(coefficients(lm2)[2],2)
        lm.smry$MDS.R2[file.i] <- round(summary(lm2)$r.squared,2)
        lm.smry$MDS.rMSE[file.i] <- round(mean.error.MDS,2)
        lm.smry$FX.bias[file.i] <- round(bias.FX,2)
        lm.smry$MDS.bias[file.i] <- round(bias.MDS,2)
        
        mean.exp <- mean(data$exp.ddG)
        TSS <- sum((data$exp.ddG - mean.exp)^2)
        
        fX.SS <- sum((data$exp.ddG - data$foldX.ddG)^2)
        fX.Resids <- (data$exp.ddG - data$foldX.ddG)
        MS.SS <- sum((data$exp.ddG - data$MD.fX.ddG)^2)
        MS.resids <- (data$exp.ddG - data$MD.fX.ddG)
        
        R2.fX <- (TSS - fX.SS)/TSS
        R2.MS <- (TSS - MS.SS)/TSS
            
        if (plot.pdf == TRUE){
            par(mar=c(5,5,4,1))
            grey.col <- "grey75"
            pt.cex <- 0.8
            leg.loc <- "bottomright"
            scale <- 1.2
            plot(data$MD.fX.ddG, data$exp.ddG, pch=24, bg="black", cex=pt.cex, xlim=c(min.b*scale, max.b*scale), ylim=c(min.b*scale, max.b*scale), xlab=expression(paste(Delta, Delta, G, " Predicted", " (kcal/mol)")), ylab=expression(paste(Delta, Delta, G, " Experimentally Observed", " (kcal/mol)")), main=systems2[file.i])
            abline(0,1, lty="solid", col=grey.col, lwd=3)
            points(data$foldX.ddG, data$exp.ddG, pch=4, bg="white", cex=pt.cex)
            abline(coefficients(lm), lty="dashed")
            abline(coefficients(lm2))
            points(data$foldX.ddG, data$exp.ddG, pch=4, bg="black")
            points(data$MD.fX.ddG, data$exp.ddG, pch=24, bg="black")
            if (file.i == 1){
                legend(leg.loc, legend=c("Perfect Fit", as.expression(bquote(paste("Ex  ", R^2==.(format(round(summary(lm)$r.squared,2), nsmall=2))))), as.expression(bquote(paste("MD ", R^2==.(format(round(summary(lm2)$r.squared,2), nsmall=2)))))), bty="n", lty=c("solid", "dashed", "solid"), col=c(grey.col,"black", "black"), pch=c(NA, 4, 24), pt.bg=c(NA, NA, "black"), lwd=c(3,1,1))
           } else{
                legend(leg.loc, legend=c(as.expression(bquote(paste("Ex  ", R^2==.(format(round(summary(lm)$r.squared,2), nsmall=2))))), as.expression(bquote(paste("MD ", R^2==.(format(round(summary(lm2)$r.squared,2),nsmall=2)))))), bty="n")
           }
        }
    }
                        
    for (col.i in 2:length(lm.smry[1,])){
        colmean <- round(mean(lm.smry[,col.i], na.rm=TRUE), round.vals[col.i])
        lm.smry[n.systems+1,col.i] <- colmean
    }
    
    lm.smry
                        
    if (plot.pdf == TRUE){
        dev.off()
    }
    
    if (print.table == TRUE){
        write.table(x=lm.smry, file=table.file, col.names=TRUE, row.names=FALSE, sep="\t")
    }
                        
    # Unpound these lines to output the results as LaTex Table 
        #library(xtable)
        #x <- xtable(lm.smry, digits=c(1,round.vals), floating=FALSE, tabular.environment="tabular", hline.after=NULL, include.rownames=FALSE, include.colnames=TRUE, caption="Folding Stability")
        #print(x, include.rownames=FALSE)
```

Out[146]:

|  | Protein | n.sites | n.mutations | FX.bias | FX.rMSE | FX.Intercept | FX.Slope | FX.R2 | MDS.bias | MDS.rMSE | MDS.Intercept | MDS.Slope | MDS.R2 |
| --- | --- | --- | --- | --- | --- | --- | --- | --- | --- | --- | --- | --- | --- |
| 1 | 1bni | 29 | 32 | -0.41 | 0.97 | 0.25 | 0.47 | 0.24 | -0.03 | 0.58 | 0.14 | 0.8 | 0.53 |
| 2 | 1bvc | 17 | 30 | 2.26 | 2.4 | 2.37 | 0.86 | 0.51 | 2.11 | 2.23 | 2.12 | 0.98 | 0.49 |
| 3 | 1hfz | 15 | 31 | 0.86 | 1.24 | 0.86 | 0.96 | 0.44 | 0 | 1.16 | 0.28 | 0.63 | 0.16 |
| 4 | 1lz1 | 20 | 36 | -0.5 | 0.82 | 0.15 | 0.44 | 0.34 | -0.21 | 0.55 | 0.13 | 0.61 | 0.5 |
| 5 | 1pin | 35 | 37 | -0.12 | 1.16 | 0.31 | 0.65 | 0.26 | 0.32 | 1.12 | 0.27 | 1.07 | 0.31 |
| 6 | 1rn1 | 17 | 19 | -0.48 | 0.55 | -0.49 | 1.03 | 0.46 | -0.42 | 0.44 | -0.44 | 1.13 | 0.7 |
| 7 | 1rtb | 10 | 190 | -0.36 | 2.09 | 0.94 | 0.26 | 0.22 | -0.27 | 1.89 | 0.92 | 0.29 | 0.19 |
| 8 | 1vqb | 32 | 43 | 0.75 | 0.87 | 0.76 | 0.08 | 0 | 0.59 | 0.76 | 0.5 | 1.56 | 0.25 |
| 9 | 1wq5 | 22 | 26 | -0.02 | 1.05 | 0.12 | 0.72 | 0.2 | -0.06 | 0.98 | -0.07 | 1.02 | 0.23 |
| 10 | 2abd | 21 | 71 | 2.07 | 2.45 | 2.29 | 0.13 | 0.01 | 1.81 | 1.95 | 1.69 | 1.26 | 0.16 |
| 11 | Means | NA | 52 | 0 | 1.36 | 0.76 | 0.56 | 0.27 | 0.38 | 1.17 | 0.55 | 0.94 | 0.35 |

Out[146]:

**pdf:** 2

## R Analysis of Ebola System¶

Here we first read in the our ME+FoldX analysis of the Ebola system. Column 1 is the mutation, column 2 is the GP-antibody binding affinity, column 3 is the GP1-GP2 dimer binding stability, column 4 is the GP12 trimer binding stability, and column 5 is the monomer folding stability.

In [147]:

```
setwd("/Users/Craig/Dropbox/Craig_Work/Project--Ebola/Jupytr/Workflow_Files")

ebov.data <- read.table(file="mayinga_foldx.txt", header=TRUE, stringsAsFactors=FALSE)
head(ebov.data)
tail(ebov.data)
```

Out[147]:

|  | mutation | antibody\_bind | dimer\_bind | trimer\_bind | monomer\_fold |
| --- | --- | --- | --- | --- | --- |
| 1 | SA32C | 3.433333e-05 | -0.0001833333 | 0.279439 | 0.216896 |
| 2 | SA32D | -0.000703 | 0.2400067 | 1.305792 | 0.241224 |
| 3 | SA32Q | 3.6e-05 | 0.1090533 | 0.36296 | 0.127038 |
| 4 | SA32K | 0.0002136667 | -0.2851333 | -0.383857 | 0.012134 |
| 5 | SA32P | 0.0001173333 | 0.24305 | 0.441061 | 0.623618 |
| 6 | SA32T | 5.633333e-05 | 0.02169667 | 0.276408 | 0.189022 |

Out[147]:

|  | mutation | antibody\_bind | dimer\_bind | trimer\_bind | monomer\_fold |
| --- | --- | --- | --- | --- | --- |
| 6455 | WD597L | 0 | 0.1922267 | 1.59762 | 0.242122 |
| 6456 | WD597R | -1.066667e-05 | 0.4601733 | 3.663291 | -0.079074 |
| 6457 | WD597V | 0 | 0.1073633 | 2.826126 | 0.564259 |
| 6458 | WD597N | 0 | 0.15771 | 2.519828 | 0.253396 |
| 6459 | WD597Y | 0 | 0.07497333 | 0.561132 | -0.018686 |
| 6460 | WD597M | 0 | 0.1295333 | 0.55355 | 0.0802 |

##### Define stability thresholds¶

In [148]:

```
fold.cut <- 3.0  #2.5
bind.cut <- 3.0  #2.5
anti.bind.cut <- 2.0
```

##### Identify Watch List Mutations¶

In [149]:

```
fold.via <- which(ebov.data$monomer_fold < fold.cut)
fold.invia <- which(ebov.data$monomer_fold >= fold.cut)
anti.bind.via <- which(ebov.data$antibody_bind < anti.bind.cut)
anti.bind.invia <- which(ebov.data$antibody_bind >= anti.bind.cut)
dimer.bind.via <- which(ebov.data$dimer_bind < bind.cut)
dimer.bind.invia <- which(ebov.data$dimer_bind >= bind.cut)
trimer.bind.via <- which(ebov.data$trimer_bind < bind.cut)
trimer.bind.invia <- which(ebov.data$trimer_bind >= bind.cut)
watch.2 <- watches <- intersect(fold.via, anti.bind.invia)

ebov.data[watch.2,]
```

Out[149]:

|  | mutation | antibody\_bind | dimer\_bind | trimer\_bind | monomer\_fold |
| --- | --- | --- | --- | --- | --- |
| 4728 | ND506W | 3.396693 | 0.03833 | 0.000899 | -0.411212 |
| 4730 | ND506Y | 2.564015 | 0.09399 | 0.000958 | -0.552953 |
| 4854 | PD513H | 2.520048 | 0.00507 | 0 | 0.945291 |
| 4860 | PD513W | 2.189683 | 0.00567 | 0 | 0.859639 |
| 5552 | ND550Q | 3.755913 | 0.01326667 | 6e-06 | 0.918908 |
| 5553 | ND550K | 4.5929 | 0.007086667 | 6e-06 | 0.62136 |
| 5554 | ND550P | 3.820577 | 0.14025 | 6e-06 | 2.196727 |
| 5556 | ND550F | 10.01182 | 0.02879333 | 6e-06 | 2.085338 |
| 5558 | ND550H | 5.498877 | 0.02724667 | 6e-06 | 1.805071 |
| 5560 | ND550I | 5.278627 | 0.02154667 | 0 | 1.664637 |
| 5561 | ND550E | 3.494727 | -0.04264333 | 0 | 1.142505 |
| 5563 | ND550R | 5.339023 | -0.02773 | 0 | 0.979954 |
| 5564 | ND550W | 13.52022 | 0.02061 | 0 | 2.287674 |
| 5565 | ND550V | 2.078363 | 0.01602667 | 0 | 1.736291 |
| 5566 | ND550Y | 13.51595 | 0.04176 | 0 | 1.978029 |
| 5567 | ND550M | 3.293076 | 0.01540667 | 0 | -0.149612 |
| 5588 | DD552S | 2.100565 | 0.7474833 | -0.000172 | 0.333868 |
| 5589 | DD552Q | 2.190444 | 0.3596867 | -0.000172 | 0.288601 |
| 5590 | DD552K | 2.610536 | 0.2821 | -0.000188 | 0.163696 |
| 5592 | DD552T | 2.399138 | 1.010887 | -0.000172 | 1.47302 |
| 5593 | DD552F | 4.105533 | 0.42594 | -0.000172 | 0.136739 |
| 5594 | DD552A | 2.171218 | 0.7125433 | -0.000172 | 0.484719 |
| 5595 | DD552H | 4.528623 | 0.55405 | -0.000172 | 0.294558 |
| 5596 | DD552G | 2.608564 | 0.3858367 | -0.000172 | 0.013126 |
| 5600 | DD552R | 3.299119 | 0.25924 | -0.000439 | 0.336742 |
| 5601 | DD552W | 5.05073 | 0.41288 | -0.000172 | 0.611765 |
| 5602 | DD552V | 2.411182 | 0.7460033 | -0.000172 | 1.946828 |
| 5604 | DD552Y | 4.713363 | 0.422 | -0.000172 | 0.1255 |
| 5624 | GD553M | 8.773623 | -0.01026 | 2e-06 | 2.936865 |
| 5689 | GD557F | 2.25867 | 0.13369 | 0.000173 | -1.337602 |
| 5691 | GD557H | 3.71904 | 0.6695367 | 0.000706 | -0.050353 |
| 5695 | GD557R | 2.287351 | 0.1708933 | -0.001501 | -0.617742 |
| 5696 | GD557W | 3.205722 | 0.6940733 | 0.000485 | -1.323157 |
| 5699 | GD557Y | 2.811327 | 0.1417467 | -0.000102 | -1.188949 |

In [150]:

```
#  Some other useful tid bits.
di.tri.via <- intersect(dimer.bind.via, trimer.bind.via)
di.tri.fold.via <- intersect(di.tri.via, fold.via)
via.anti.disrupt <- intersect(di.tri.fold.via, anti.bind.invia)
```

##### Plotting¶

Note code right now outputs plot to this notebook. To instead create a pdf, unpound the first and last lines of the 2nd cell below.

In [151]:

```
mon.fold <- ebov.data$monomer_fold  #[mon.cex.ord]
di.bind <- ebov.data$dimer_bind		#[mon.cex.ord]
tri.bind <- ebov.data$trimer_bind	#[mon.cex.ord]
anti.bind <- ebov.data$antibody_bind	#[mon.cex.ord]

mon.cex <- rep(1, length(mon.fold))
mon.fold.big <- which(mon.fold > 5)
mon.cex[mon.fold.big] <- 1+mon.fold[mon.fold.big]/5
mon.cex.ord <- order(mon.cex, decreasing=TRUE)

obs <- which(ebov.data$mutation[watch.2]=="ND550K")
```

In [152]:

```
#pdf(file="watch_list_figure_v5.pdf", width=6, height=6)
par(mar=c(5,5,4,1))
let.cex <- 1.2
label.cex <- 1.3
plot(anti.bind, max.ddG, pch=20+max.id, bg="white", xlim=c(-2, 15), ylim=c(-3, 15), xlab=expression(paste(Delta, Delta, "G"["Antibody-GP binding"], " (kcal/mol)")), ylab=expression(paste(Delta, Delta, "G"["max"], " (kcal/mol)")), cex.lab =label.cex, xaxt="n", yaxt="n")
axis(side=1, at=c(seq(0,5),10,15), labels=c(0,rep(NA, 4),5,10,15))
axis(side=2, at=c(seq(0,5),10,15), labels=c(0,rep(NA, 4),5,10,15))
#abline(0,0)
#abline(v=0)
abline(3.0, 0, lty="dashed")
abline(v=2.0, lty="dashed")
abline(-3.0, 0, lty="dashed")
#abline(v=-2.5, lty="dashed")
points(anti.bind[watches], max.ddG[watches], pch=20+max.id[watches], bg="red", cex=1.2)
text(-1.5,-1.5, labels="A", font=2, cex=let.cex)
text(-1.5,10, labels="B", font=2, cex=let.cex)
text(3.9, 10, labels="C", font=2, cex=let.cex)
text(3.9,-1.5, labels="D", font=2, col="black", cex=let.cex)
text(9,0, labels="Watch List", font=2, col="red", cex=let.cex)
points(anti.bind[watches[obs]], max.ddG[watches[obs]], pch=20+max.id[watches], bg="red", cex=1.75)
text(anti.bind[watches[obs]], max.ddG[watches[obs]], labels="N550K", adj=c(0,3))
points(c(anti.bind[watches[obs]],anti.bind[watches[obs]]+0.3), c(max.ddG[watches[obs]], max.ddG[watches[obs]]-1), type="l")
legend(9, 11, legend=c("Dimer binding", "Trimer binding", "Monomer folding"), pch=c(21, 22, 23), pt.bg="white", cex=1.0, bg="white")

#text(anti.bind[watches], max.ddG[watches], labels=letters[1:24], pos=4, cex=0.75)

#dev.off()
```

xml version="1.0" encoding="UTF-8"?
