# Supplementary figures and images for: Initiating a watch list for Ebola virus antibody escape mutations
Source: PeerJ. 2016 Feb 16;4:e1674. doi: 10.7717/peerj.1674 (PMC4768679; doi:10.7717/peerj.1674)

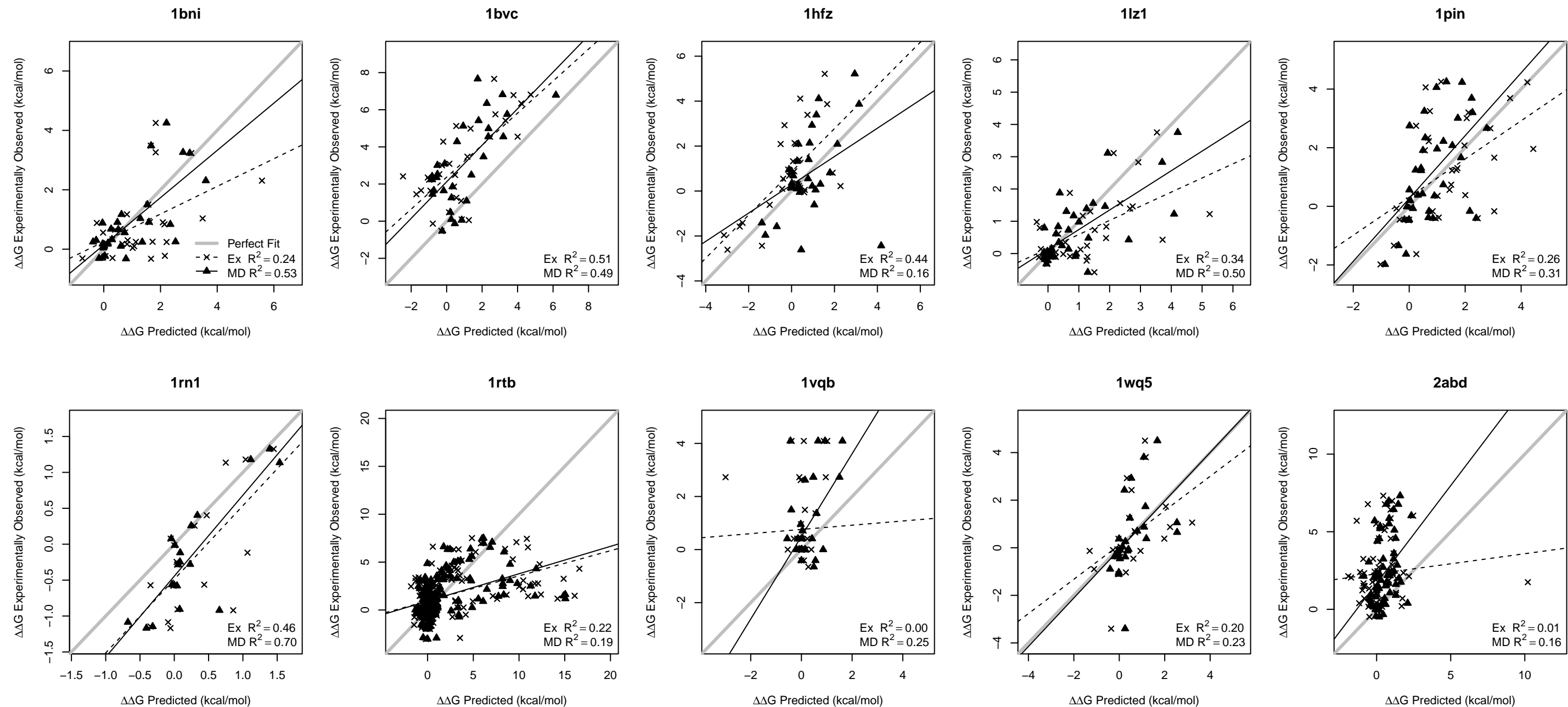

Supplement: Supplemental Information 3 [file peerj-04-1674-s003.zip › Workflow_files_zip/test_systems/Binding ddG experimental systems (v2).pdf]

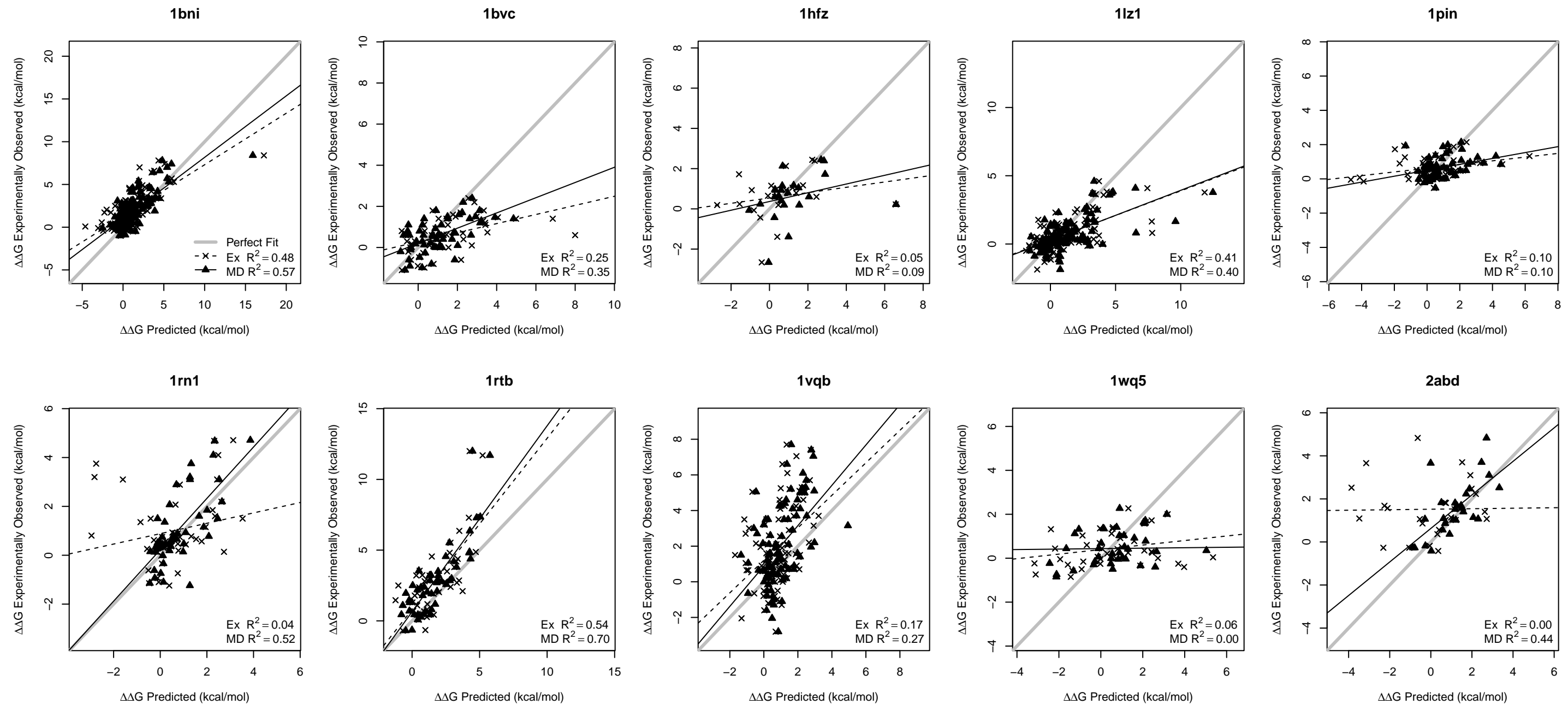

Supplement: Supplemental Information 3 [file peerj-04-1674-s003.zip › Workflow_files_zip/test_systems/Folding ddG experimental systems (v2).pdf]

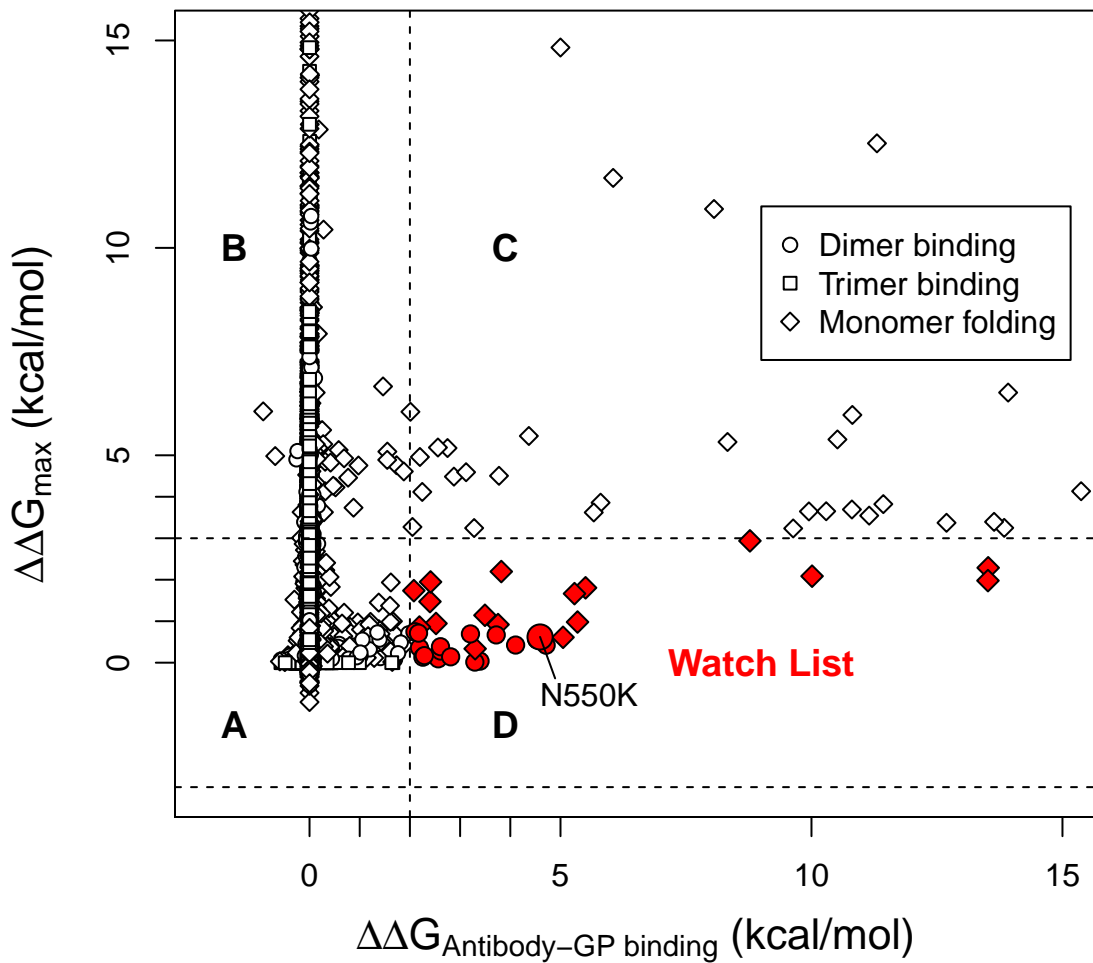

Supplement: Supplemental Information 3 [file peerj-04-1674-s003.zip › Workflow_files_zip/watch_list_fig_v5.pdf]
